# The serological responses to acute exercise in humans reduce cancer cell growth in vitro: A systematic review and meta‐analysis

**DOI:** 10.14814/phy2.14635

**Published:** 2020-11-18

**Authors:** Samuel T. Orange, Alastair R. Jordan, John M. Saxton

**Affiliations:** ^1^ School of Biomedical, Nutritional, and Sport Sciences Faculty of Medical Sciences The Medical School Newcastle University Newcastle upon Tyne UK; ^2^ School of Sport York St John University York UK; ^3^ Department of Sport, Exercise and Rehabilitation Faculty of Health and Life Sciences Northumbria University Newcastle upon Tyne UK

**Keywords:** biomarkers, cancer prevention, exercise physiology, physical activity, serum

## Abstract

We systematically reviewed and meta‐analyzed the effects of acute exercise‐conditioned serum on cancer cell growth in vitro. Five literature databases were systematically searched for studies that measured cancer cell growth after exposure to human sera obtained before and immediately after an acute bout of exercise. Standardized mean differences (SMDs) with 95% confidence intervals (CIs) were pooled using a three‐level random‐effects model. Meta‐regressions were also performed with participant age and disease status, exercise type, cell line *TP53* status, and serum incubation time entered as covariates. Seven studies met the inclusion criteria encompassing a total of 21 effect estimates and 98 participants. Exercise‐conditioned serum significantly reduced cancer cell growth compared with preexercise serum (SMD = −1.23, 95% CI: −1.96 to −0.50; *p* = .002; *I* ^2^ = 75.1%). The weighted mean reduction as a percentage of preexercise values was 8.6%. The overall treatment effect and magnitude of heterogeneity were not statistically influenced by any covariate. There were concerns regarding the risk of bias within individual studies and Egger's test of the intercept showed evidence of small study effects (*β* = −3.6, *p* = .004). These findings provide in vitro evidence that the transient serological responses to acute exercises reduce cancer cell growth, although many questions remain regarding the underlying mechanistic pathways and potential effect modifiers. To strengthen this evidence‐base, future studies should seek to reduce the risk of bias by using more rigorous experimental designs, and consider using 3D cell culture models to better replicate in vivo tumor conditions. PROSPERO registration: CRD42020161333.

## Graphical abstract

This systematic review and meta‐analysis showed that exercise‐conditioned serum significantly reduces in vitro cancer cell growth. This finding provides preliminary evidence that acute exercise‐induced modulations in serum markers may, at least in part, explain the link between regular physical activity and reduced cancer risk.

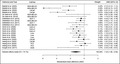

## 1 INTRODUCTION

There is a growing body of observational research linking physical activity with cancer prevention. Indeed, strong epidemiological evidence shows that regular physical activity reduces the risk of developing several cancer types including colon, postmenopasual breast, and endometrial (Physical Activity Guidelines Advisory Committee Scientific Report, 2018; World Cancer Research Fund/American Institute for Cancer Research, 2018a). Physical activity after a cancer diagnosis is also associated with reduced risk of cancer recurrence and mortality in colon, breast, and prostate cancer (Cormie et al., 2017; Patel et al., 2019). This body of evidence has given rise to cancer‐specific physical activity guidelines, with the World Cancer Research Fund/American Institute for Cancer Research (2018b) recommending that individuals engage in at least 150 min wk^−1^ of moderate‐intensity or 75 min wk^−1^ of vigorous‐intensity aerobic physical activity to reduce cancer risk (World Cancer Research Fund/American Institute for Cancer Research, 2018b).

The risk‐reducing effect of physical activity is mainly thought to occur through changes in circulating biomarkers related to cancer risk, including modulations in endogenous insulin, inflammatory mediators, sex hormones, adipokines, and insulin‐like growth factor 1 (IGF‐1) (Friedenreich et al., 2017; McTiernan, 2008). However, intervention studies show that these changes are largely dependent on reductions in body fat (Batacan et al., 2017; Dethlefsen, Pedersen, et al., 2017; Grieco et al., 2013; Kang et al., 2017; McTiernan, 2008; Sturgeon et al., 2016). Even so, most inverse associations between physical activity and cancer risk reported in the epidemiological literature remain statistically significant even after adjustment for body mass index (BMI) (Moore et al., 2016). Furthermore, a recent Mendelian randomization study using UK Biobank data showed that physical activity is inversely related to breast and colon cancer risk, independent of adiposity (Papadimitriou et al., 2020). This suggests that the antineoplastic effects of exercise are explained, at least in part, by biological pathways other than weight loss‐induced changes (indicative of reduced body fatness) in resting systemic risk factors.

Aside from the chronic adaptations that occur in response to regular physical activity, each bout of exercise results in marked yet short‐lasting alterations in many circulating serum factors. Acute serological responses to exercise include the release of catecholamines, anti‐inflammatory cytokines and myokines. (Petersen & Pedersen, 2005; Zouhal et al., 2008). Recent review papers have proposed that these repetitive acute serological responses could culminate over time to reduce cancer risk (Christensen et al., 2018; Dethlefsen, Pedersen, et al., 2017; Hojman et al., 2018), which has important implications for exercise prescription in this context. The experimental model used to evaluate this theory involves stimulating a cancer cell line with serum collected before and after an acute bout of exercise. However, to date, no study has systematically synthesized nor critically appraised the original research studies that have explored the impact of acute exercise on cancer cell growth. Therefore, the purpose of this study was to systematically review and meta‐analyze the effects of acute exercise‐conditioned serum on cancer cell growth in vitro.

## 2 METHODS

This review was prospectively registered in the PROSPERO prospective register of systematic reviews (ref: CRD42020161333) and followed the Preferred Reporting Items for Systematic Reviews and Meta‐Analyses (PRISMA) guidelines (Shamseer et al., 2015).

### 2.1 Search strategy

An electronic search of PubMed, Web of Science, Scopus, SportDiscus, and CINAHL was conducted from inception to 3rd July 2020. The specific search terms we used are presented in Table 1. We also manually screened the reference lists and performed forward citation of included studies to identify potentially eligible studies.

**TABLE 1:** Search terms used in PubMed

[All fields] Cancer OR neoplas* OR malignan* OR carcinoma OR tumor OR tumour
AND
[All fields] Exercise OR "physical activity" OR aerobic OR anaerobic OR "resistance training" OR “strength training” OR “weight training”
AND
[All fields] Cells OR “cell line*” OR “in vitro”
AND
[All fields] Serum OR sera
AND
[All fields] Growth OR proliferation OR “viability” OR “cell number”

### 2.2 Inclusion criteria

Inclusion criteria were: (a) original research articles published in a peer‐reviewed journal, (b) full‐text was available in English, (c) sera were obtained from human participants before and immediately after (≤5 min) an acute bout of exercise, (d) pre and postexercise sera were used to stimulate an established human cancer cell line, (e) serum‐stimulated cancer cell growth was measured after a period of incubation, and (f) reported data were sufficient to include in a meta‐analysis. We defined exercise as a planned, structured activity requiring physical effort, carried out to sustain or improve health and fitness.

### 2.3 Outcome measure

The primary outcome was cancer cell growth, which involved the use of cell viability or proliferation assay. Where studies used more than one cancer cell line, all relevant data were extracted and included in the meta‐analysis.

### 2.4 Study selection

Once all literature searches were complete, studies were compiled into a single list in an Excel spreadsheet (Microsoft Corporation). One author (STO) removed duplicates and screened the titles and abstracts to identify potentially relevant trials. Full‐texts were then obtained for all studies that appeared to meet the inclusion criteria or where there was any uncertainty. Subsequently, two authors (STO, ARJ) independently examined each full‐text manuscript to assess for eligibility. Any disagreements were resolved through discussion and/or consultation with the third author (JMS). Corresponding authors were contacted if a full‐text manuscript could not be retrieved or to clarify aspects of the study.

### 2.5 Data extraction

Data items extracted from each eligible study included: (a) participant characteristics, (b) sample size, (c) characteristics of the acute exercise bout, (d) type of cancer studied, (e) details of the cell growth assay, (f) details of the serum markers and signaling pathways evaluated, and (g) cell growth data (mean ± *SD*). Study authors were contacted to obtain missing data wherever necessary. If data were only presented graphically and we did not receive a response from study authors, we used a web‐based digitizing tool to extract the data from graphs (WebPlotDigitizer) (Drevon et al., 2017). Where the same study data were reported in multiple manuscripts, the data were only extracted from the manuscript with the earliest publication date. All data were extracted independently by two authors (STO, ARJ) and tabulated in custom‐designed Excel spreadsheets. Review authors cross‐checked coding sheets and resolved any discrepancies with discussion and consensus.

### 2.6 Risk of bias

The risk of bias within individual studies was assessed with the modified Research Triangle Institute (RTI) Item Bank for cross‐sectional studies (Viswanathan & Berkman, 2012). The original item bank consists of 29 items covering eleven different domains: sample definition and selection, interventions/exposure, outcomes, creation of treatment groups, blinding, soundness of information, follow‐up, analysis comparability, analysis outcome, interpretation, and presentation and reporting. In line with the authors’ instructions (Viswanathan & Berkman, 2012), we removed questions that were not applicable to this review, which left a total of 14 items. For each item in a selected study, a positive response indicated a low risk of bias, a negative response indicated a high risk of bias, and an unclear risk of bias was given if a “partially” or “cannot be determined” response was observed (Margulis et al., 2014). Two authors (STO, ARJ) independently appraised each study and resolved discrepancies via discussion with the third author (JMS). Small study effects were explored with a funnel plot and Egger's test of the intercept (Egger et al., 1997).

### 2.7 Statistical analysis

To quantify the effect of serum‐stimulated cancer cell growth, standardized mean differences (SMDs) between postexercise and preexercise serum were calculated as the mean difference divided by the *SD* of the difference (SD_diff_). Hedges *g* correction was applied to adjust for sample bias. Qualitative descriptors used to interpret the strength of the SMDs were based on Cohen's (1988) criteria (±): trivial (< 0.2), small (0.2–0.49), moderate (0.5–0.79), large (≥0.8). Negative SMDs represent an exercise‐induced reduction in cell growth.

If a study did not report *SD*_diff_, and it could not be retrieved from the corresponding author, it was estimated using the *SD*s in cell viability at preexercise (*SD*_pre_) and postexercise (*SD*_post_), in addition to the correlation (*r*) between measures (Higgins et al., 2019):

$$SD_{\text{diff}}=\sqrt{SD_{\text{pre}}^{2}+SD_{\text{post}}^{2}‐\left(2\times r\times SD_{\text{pre}}\times SD_{\text{post}}\right)}$$

We followed guidelines by Rosenthal (1993) to assume a conservative correlation of 0.7. Sensitivity analyses were performed with *r* = .5 and *r* = .9 to determine whether the results were robust to the use of imputed correlations.

A meta‐analysis of SMDs was performed using a random‐effects model with a three‐level structure. SMDs were nested within studies to account for correlated effects within studies (Van den Noortgate et al., 2013). A random‐effects model was chosen because it was assumed that due to methodological differences (e.g., cancer cell line used), studies were estimating different, yet related effects (Borenstein et al., 2010). The model was fitted with the maximum likelihood estimation and studies were weighted according to the inverse of the sampling variance. Confidence intervals (CIs) were adjusted using the Knapp–Hartung adjustment due to the low number of studies (IntHout et al., 2014). The weighted mean difference as a percentage of preexercise values was also calculated.

The magnitude of heterogeneity not attributable to sampling error was using evaluated with *I* ^2^. Thresholds of *I* ^2^ were in line with Cochrane recommendations: 0%–40% (“might not be important”), 30%–60% (“may represent moderate heterogeneity”), 50%–90% (“may represent substantial heterogeneity”), and 75%–100% (“considerable heterogeneity”) (Deeks et al., 2019). The importance of the *I* ^2^ value was interpreted alongside the *p* value from the Chi‐squared test (Deeks et al., 2019). Sources of heterogeneity were explored using random‐effects meta‐regressions with the mean age of participants (continuous variable), type of aerobic exercise (interval vs. continuous exercise), cancer type (breast vs. other cancer), disease status of participants (cancer survivor vs. apparently healthy), *TP53* gene status of the cancer cell line (mutated vs. wild type), and serum incubation time (≥72 hr vs. ≤48 hr) entered into the model as covariates. We chose to explore the potential moderating effect of *TP53* status because somatic mutation to the *TP53* gene is the most common mutation across all cancers and *TP53* status is well characterized among cancer cell lines (Leroy et al., 2014).

Statistical analyses were conducted in R version 3.5.2 (R Foundation for Statistical Computing). Statistical significance was set at *p* < .05. Data are presented as SMD with corresponding 95% CIs. The search results, dataset, and statistical code are available on Open Science Framework (XXXX, 2020).

## 3 RESULTS

### 3.1 Study selection

**FIGURE 1:**
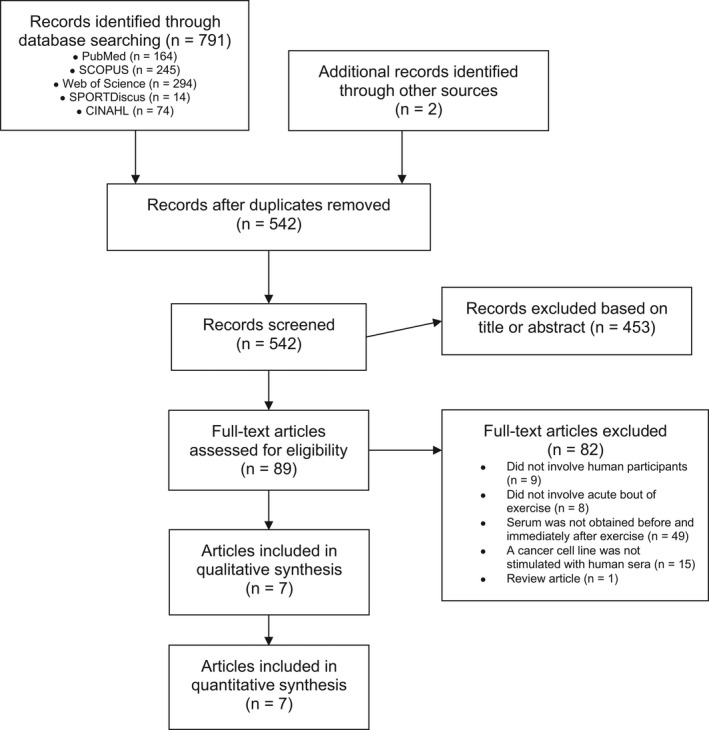
Preferred Reporting Items for Systematic Reviews and Meta‐Analyses (PRISMA) flow diagram of the systematic search and included studies

The primary search yielded a total of 791 abstracts, of which 249 were duplicates (Figure 1). After screening the titles and abstracts, a further 453 abstracts were removed and 89 full‐texts were then retrieved and assessed for eligibility. Seven manuscripts involving a total of 98 participants met the inclusion criteria and were included in this review and meta‐analysis. The median sample size was 10 (range: 3–20).

### 3.2 Study and exercise characteristics

The included studies involved breast (MDA‐MB‐231, MCF‐7) (Baldelli et al., 2020; De Santi et al., 2019; Dethlefsen, Hansen, et al., 2017; Dethlefsen et al., 2016) colon (CaCo‐2, LoVo) (Devin et al., 2019), lung (A549) (Kurgan et al., 2017), and prostate (LNCaP, PC3) (Baldelli et al., 2020; Hwang et al., 2020) cancer cell lines (Table 2). Cell lines differed with respect to their mutation status of cancer critical genes including TP53, KRAS, and BRAF. Two studies recruited cancer survivors (Dethlefsen et al., 2016; Devin et al., 2019) whereas the other five studies recruited apparently healthy adults (Baldelli et al., 2020; De Santi et al., 2019; Dethlefsen, Hansen, et al., 2017; Hwang et al., 2020; Kurgan et al., 2017). All studies involved aerobic exercise; four studies involved high‐intensity aerobic interval exercise on a cycle ergometer, (Baldelli et al., 2020; Dethlefsen et al., 2016; Devin et al., 2019; Kurgan et al., 2017) and the remaining three studies involved continuous aerobic exercise on a cycle ergometer (Dethlefsen, Hansen, et al., 2017; Hwang et al., 2020) or treadmill (De Santi et al., 2019). One study also combined aerobic exercise with resistance training (Dethlefsen et al., 2016).

**TABLE 2:** Description of included studies

Author	Participants	Age (years)	Exercise characteristics	Cancer type (cell line)	Serum markers	Signaling pathways^a^	Incubation time (hrs)	Cell growth measurement
Baldelli et al. (2020)	Healthy, sedentary males (*n* = 18) and females (*n* = 12)	M: 21 ± 1 F: 21 ± 1	High‐intensity aerobic interval exercise on a cycle ergometer ‐ 2 min warm‐up ‐ 20 min @ 50%–70% WR_max_ ‐ 10 × 90 s @ 90% WR_max_ separated by 3 min @ 55% WR_max_	Breast (MDA‐MB‐231) and prostate (LNCaP)	↑ CK	↔ p‐YAP ↔ p‐GSK3β	48	Haemocytometer
Devin et al. (2019)	Male colorectal cancer survivors (*n* = 10)	64 ± 6	High‐intensity aerobic interval exercise on a cycle ergometer ‐ 10 min warm‐up @ 50%–70% HR_max_ ‐ 4 × 4 min @ 85%–95% HR_max_ separated by 3 min active recovery	Colon (CaCo‐2, LoVo)	↑ IL‐6 ↑ IL‐8 ↑ TNF‐α ↑ Insulin ? IGF‐1 ? Glucose	‐	24, 48, and 72	Fluorescence via the Alamar Blue assay
De Santi et al. (2019)	Healthy, sedentary, premenopausal women (*n* = 3)	43 ± 10	Aerobic exercise on a treadmill ‐ 20 min @ 42% HRR ‐ 45 min @ 60% HRR	Breast (MDA‐MB‐231)	‐	‐	72	Fluorescence via the CellTiter 96 Aqueous Nonradioactive Cell Proliferation assay
Dethlefsen et al. (2016)	Female breast cancer survivors receiving adjuvant chemotherapy (*n* = 20)	50 ± 7	Resistance training and high‐intensity aerobic interval exercise ‐ 30 min dynamic warm‐up combined with balance training ‐ 45 min resistance training consisting of three sets of 5–8 repetitions of six resistance exercises performed @ 70%–100% 1RM ‐ 15 min aerobic interval training on a cycle ergometer @ 85%–95% HR_max_	Breast (MCF‐7, MDA‐MB‐231)	↑ IL‐6 ↑ IL‐8 ↔ IL‐10 ↑ TNF‐α ↑ EPI ↑ NEPI ↑ Lactate ↔ Insulin	‐	48	Fluorescence via the CellTiter‐Fluor assay
Dethlefsen et al. (2017)	Healthy women aged 18–30 years (*n* = 7)	25 ± 1	Aerobic exercise on a cycle ergometer ‐ 120 min @ 55% VO_2peak_	Breast (MCF‐7, MDA‐MB‐231)	↑ IL‐6 ↑ EPI ↔ *N*‐EPI ↑ Lactate	↑ p‐YAP (MCF‐7 only) ↔ t‐YAP ↔ t‐TAZ	48	Fluorescence via the CellTiter‐Fluor assay
Hwang et al. (2020)	Young (*n* = 12) and older (*n* = 10) males	Y: 28 ± 3 O: 63 ± 7	Aerobic exercise on a cycle ergometer ‐ 20 min @ 50% VO_2max_ ‐ 40 min @ 65% VO_2max_	Prostate (LNCaP, PC3)	↑ ON ↑ OSM ↔ IL‐6 ↔ IL‐15 ↑Test (Y only)	‐	96	Fluorescence via the Alamar Blue assay
Kurgan et al. (2017)	Male, recreationally active University students (*n* = 6)	22 ± 2	High‐intensity aerobic interval exercise on a cycle ergometer ‐ 4 min warm‐up ‐ 6 × 1 min @ WR_max_, separated by 1 min active recovery	Lung (A549)	↔ Insulin	↓ p‐AKT ↓ p‐mTOR ↓ p‐S6K1 ↓ p‐ERK1/2 ↔ t‐AKT ↔ t‐mTOR ↔ t‐S6K1 ↔ t‐ERK1/2	72	Absorbance via the Crystal Violet Staining assay

Abbreviations: ?, not reported; ↑, statistically significant increase from pre to postexercise; ↔, no evidence of an effect; AKT, Protein Kinase B; CK, creatine kinase; EPI, epinephrine; ERK1/2, extracellular signal‐regulated kinase 1/2; F, females; GSK3β, glycogen synthase kinase 3 beta; HR_max_, age‐predicted maximum heart rate; HRR, age‐predicted heart rate reserve; IL, interleukin; M, males; mTOR, mammalian target of rapamycin; *N*‐EPI, norepinephrine; O, older; ON, osteonectin; OSM, oncostatin M; p, phosphorylated; S6K1, ribosomal protein S6 kinase beta‐1; t, total; TAZ, transcriptional coactivator with PDZ‐binding motif; Test, testosterone; TNF‐α, tumor necrosis factor‐alpha; VO_2max_, maximal oxygen consumption; VO_2peak_, peak oxygen consumption; WR_max_, maximum work rate; Y, young; YAP, Yes‐associated protein.

^a^ The evaluation of signaling pathways in Dethlefsen, et al. (2017) was performed with serum obtained from breast cancer survivors in the previous study from the same group Dethlefsen et al. (2016).

### 3.3 Heterogeneity and risk of bias

**FIGURE 2:**
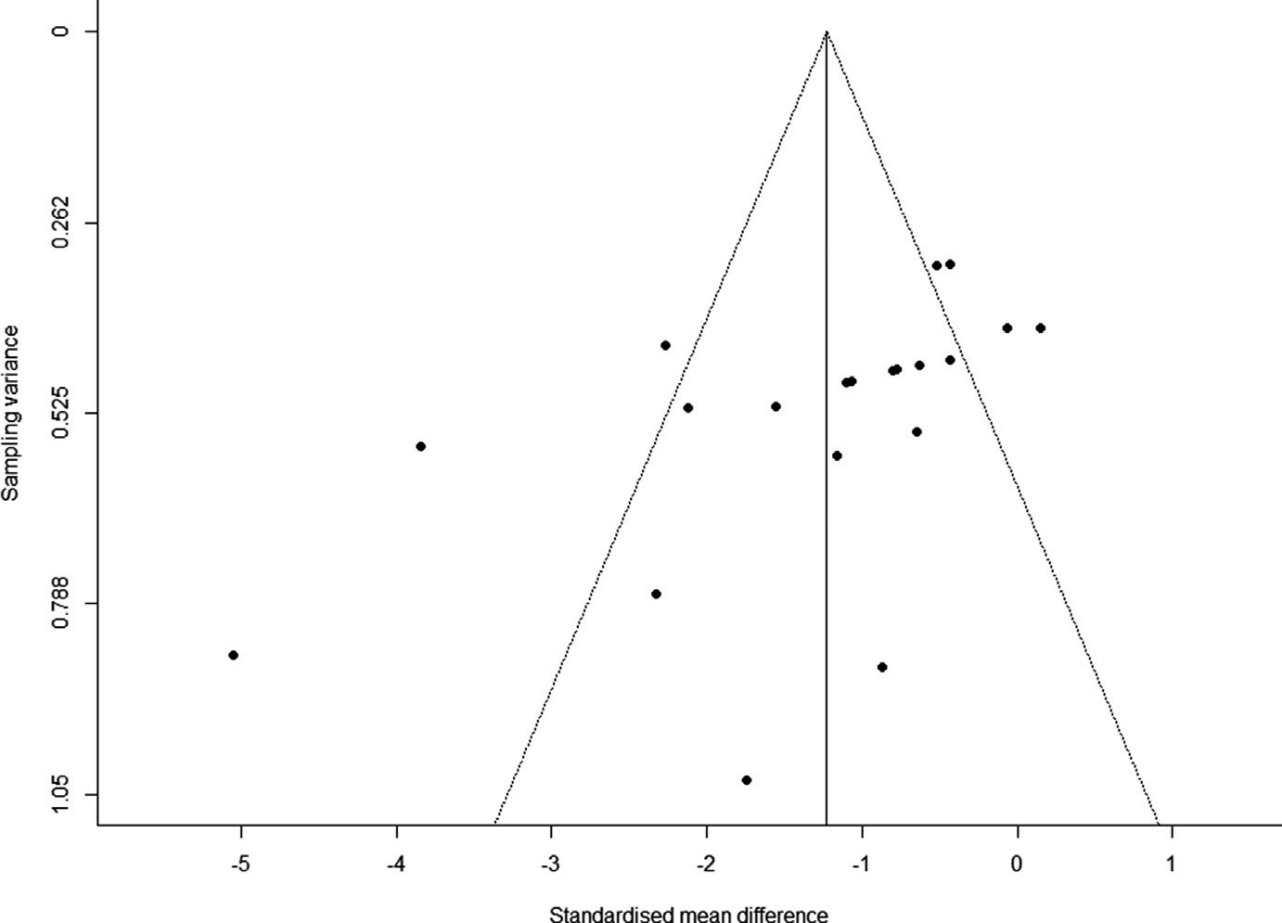
Funnel plot of the standardized mean differences from individual studies against the corresponding sampling variances

The total amount of heterogeneity was considerable (*I* ^2^ = 75.1%, *p* < .001). Visual inspection of the funnel plot showed some asymmetry (Figure 2), and Egger's test of the intercept was statistically significant (*β* = −3.6, 95% CI: −5.9 to −1.3; *p* = .004).

**FIGURE 3:**
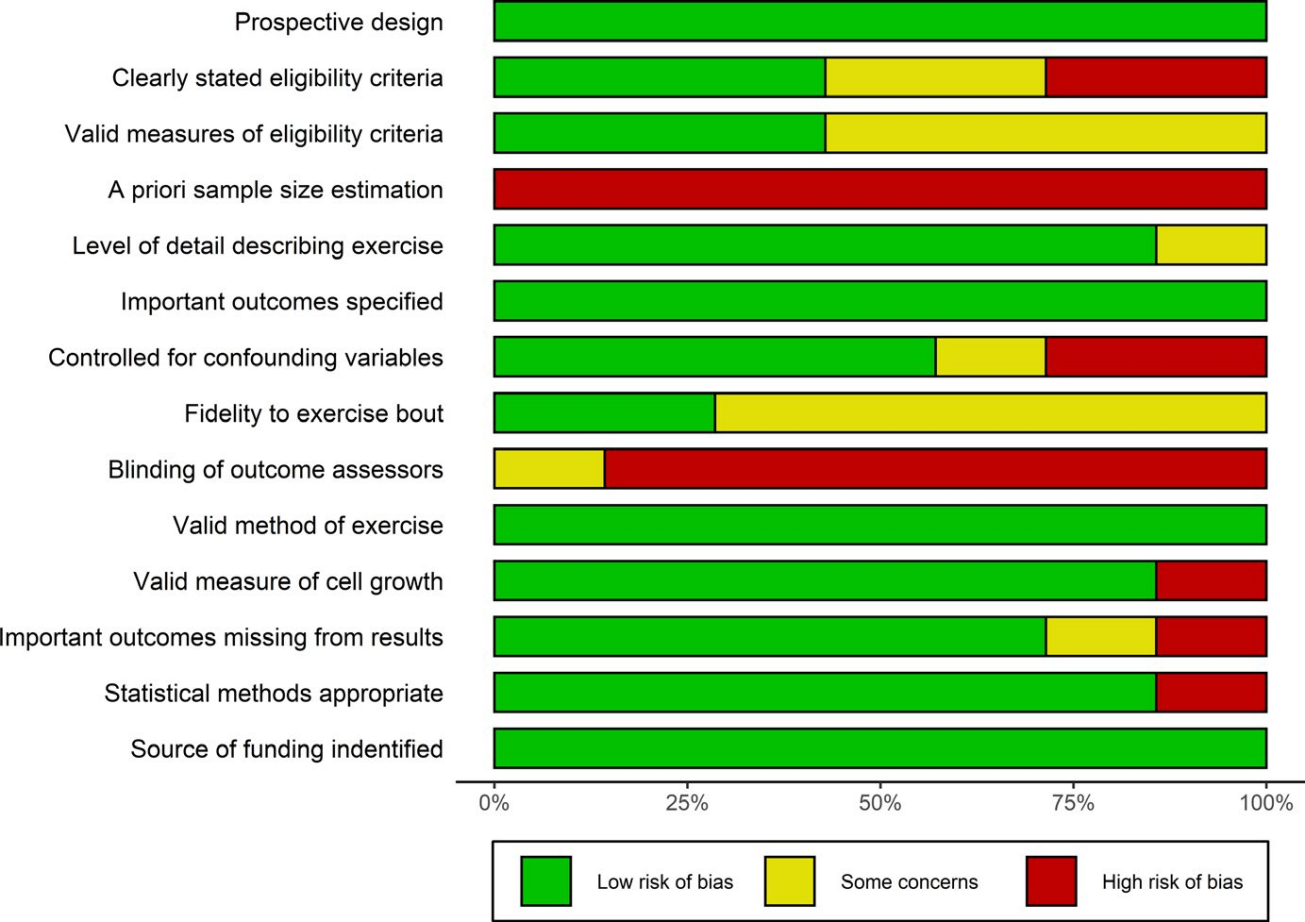
Risk of Bias plot illustrating the proportion of included studies with each bias rating (low, high, or unclear risk) for each item in the RTI Bank Tool

The risk of bias within individual studies is summarized in Figure 3 and described in detail elsewhere (XXXX, 2020). No studies performed an a priori sample size estimation. Only one study (partially) blinded outcome assessors (Devin et al., 2019), fidelity to the exercise protocol was unclear in five out of the seven studies (Baldelli et al., 2020; De Santi et al., 2019; Dethlefsen et al., 2016; Hwang et al., 2020; Kurgan et al., 2017) and only four studies were considered to adequately control for potentially confounding variables such as diet and physical activity prior to the experimental session (Baldelli et al., 2020; Dethlefsen, Hansen, et al., 2017; Hwang et al., 2020; Kurgan et al., 2017).

### 3.4 Meta‐analysis results

**FIGURE 4:**
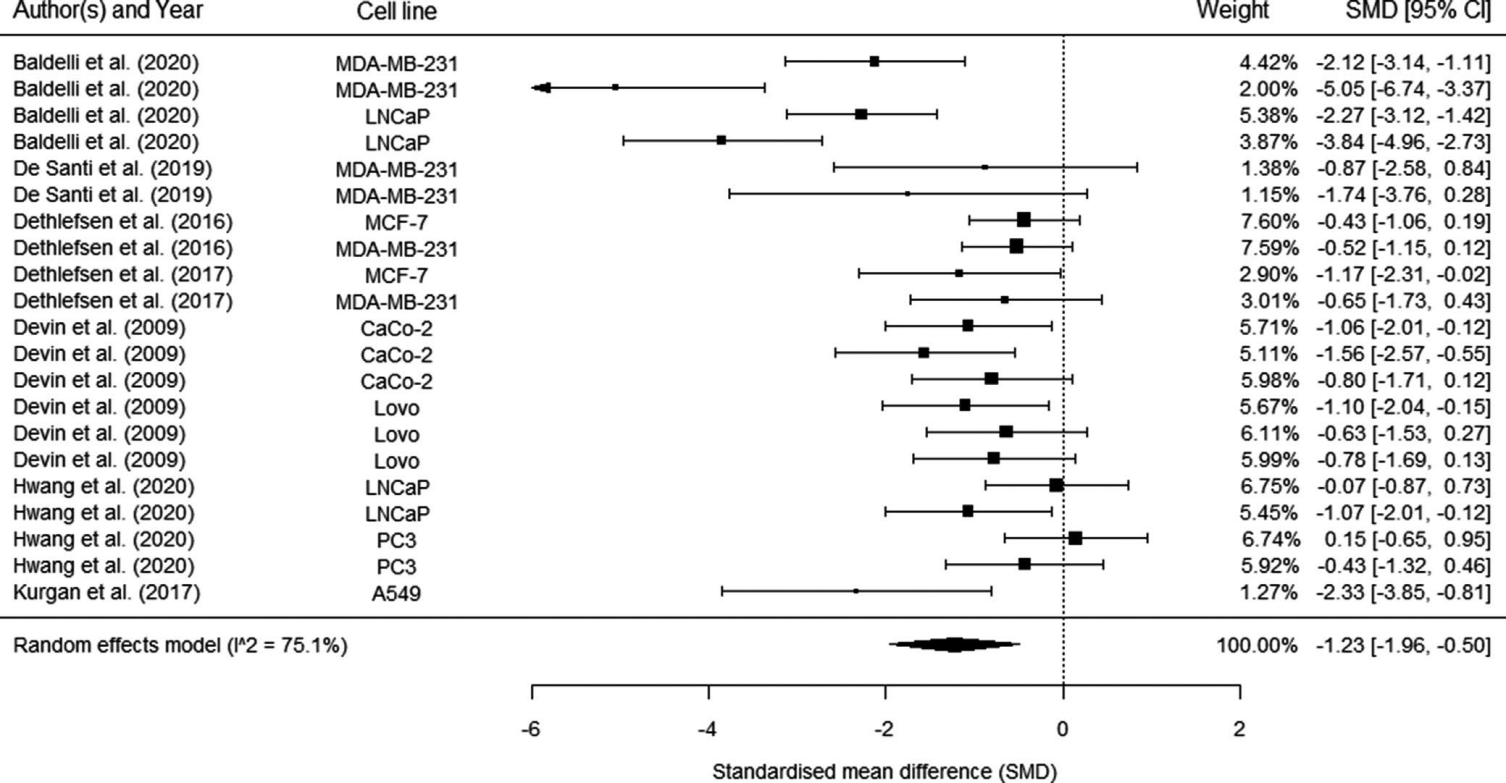
Forest plot of the results from a three‐level random‐effects meta‐analysis on the effects of exercise‐conditioned serum on cancer cell growth. Data are presented as standardized mean differences (SMDs) between pre and postexercise timepoints with corresponding 95% confidence intervals (CIs)

The meta‐analysis comprised of 21 effect estimates from seven studies (Figure 4). Exercise‐conditioned serum significantly reduced cancer cell growth compared with preexercise serum (SMD = −1.23, 95% CI: −1.96 to −0.50; *p* = .002). The weighted mean reduction as a percentage of preexercise values was 8.6%.

### 3.5 Sensitivity analyses

The SD_diff_ was unavailable for extraction from three studies (Dethlefsen, Hansen, et al., 2017; Dethlefsen et al., 2016; Kurgan et al., 2017). Sensitivity analyses showed that estimating the SD_diff_ assuming *r* = 0.5 (SMD = −1.12, 95% CI: −1.85 to −0.41; *p* = .004; *I* ^2^ = 74.8%) or *r* = 0.9 (SMD = −1.49, 95% CI: −2.30 to −0.69; *p* = .001; *I* ^2^ = 79.8%) instead of *r* = .7 did not change the conclusions of the meta‐analysis.

### 3.6 Meta‐regressions

Inclusion of study characteristics as covariates in the meta‐analysis model had a negligible influence on the magnitude of heterogeneity and did not change the outcome in such a way that the 95% CI of the SMD crossed the line of no effect (Table 3).

**TABLE 3:** Meta‐regression results

Covariate	Coefficient (95% CI)	*p* value	*I* ^2^ (*χ* ^2^ *p* value)
Age	−0.01 (−0.03 to 0.01)	0.37	79.0% (<0.001)
Cancer type			
Breast	0.08 (−0.88 to 1.0)	0.87	74.9% (<0.001)
Other	—	—	—
Aerobic exercise type			
Interval exercise	−0.82 (−2.2 to 0.53)	0.22	70.7% (<0.001)
Continuous exercise	—	—	—
Disease status			
Cancer survivor	0.75 (−0.70 to 2.2)	0.29	71.9% (<0.001)
Apparently healthy	—	—	—
*TP53* gene status			
Mutated	0.05 (−0.42 to 0.51)	0.83	75.0% (<0.001)
Wild type	—	—	—
Incubation time			
≤48 hr	0.28 (−0.48 to 1.0)	0.44	73.8% (<0.001)
>48 hr			

Abbreviations: 95% CI, 95% confidence interval; *χ* ^2^, Chi‐squared test.

## 4 DISCUSSION

The results of our systematic review and meta‐analysis show that exercise‐conditioned serum reduces in vitro cancer cell growth. This finding provides preliminary evidence that the serological responses to acute bouts of exercise may, at least in part, explain the link between regular physical activity and reduced cancer risk.

Based on the pooled data from seven studies, stimulating cancer cell lines with serum obtained immediately after a bout of exercise reduced cell growth by 8.6% compared with preexercise serum. This finding provides preliminary corroboration for the theory that repetitive spikes in circulating factors in response to acute bouts of exercise could culminate over time to reduce cancer risk and tumor growth (Dethlefsen, Pedersen, et al., 2017). The modest magnitude of the growth inhibition may be because it reflects only one exercise bout. In cases where exercise is repeated multiple times each week over an extended period, the exercise‐induced serological factors have multiple opportunities to interact with dysplastic or cancerous cells, culminating in a more pronounced suppression of cellular growth (Dethlefsen, Pedersen, et al., 2017; Hojman et al., 2018). This suggests that being physically active on several days per week, rather than accumulating all weekly activity across one or two days per week, may be particularly important for reducing cancer risk. While speculative, this proposition aligns with current physical activity guidance recommending that adults are physically active every day to elicit optimal health benefits (Physical Activity Guidelines Advisory Committee Scientific Report, 2018; World Cancer Research Fund/American Institute for Cancer Research, 2018b).

Six included studies evaluated exercise‐induced changes in serological biomarkers in attempts to identify which physiological factors were associated with changes in cancer cell growth (Baldelli et al., 2020; Dethlefsen, Hansen, et al., 2017; Dethlefsen et al., 2016; Devin et al., 2019; Hwang et al., 2020; Kurgan et al., 2017). Serum epinephrine, norepinephrine, pro‐inflammatory cytokines (TNF‐α), and myokines (IL‐6, IL‐8, osteonectin, oncostatin M) were reported to significantly increase following acute bouts of exercise (Dethlefsen, Hansen, et al., 2017; Dethlefsen et al., 2016; Devin et al., 2019; Hwang et al., 2020). While the underlying mechanistic pathways linking these serological factors to reduced cancer progression are not fully understood, there is accumulating evidence that IL‐6 release from skeletal muscle during exercise may play an important role, either directly or indirectly through the induction of anti‐inflammatory cytokines such as IL‐1 receptor antagonist (IL‐1ra) (Christensen et al., 2018; Petersen & Pedersen, 2005). During exercise, IL‐6 signaling stimulates the release of IL‐1ra from macrophages and monocytes, leading to elevated levels in serum (Gleeson et al., 2011; Steensberg et al., 2003). IL‐1ra competitively blocks the pro‐inflammatory actions of IL‐1α and IL‐1β at the receptor level, which have been implicated in tumor growth (Apte et al., 2006). However, while IL‐1ra administration has been shown to inhibit tumor growth in vivo, several studies have failed to show an effect of IL‐1ra treatment on proliferation rates in vitro (Elaraj et al., 2006; Lewis et al., 2006; Triozzi et al., 2011).

Administration of IL‐6 and oncostatin M has been shown to suppress breast cancer growth in vitro (Chiu et al., 1996; Hojman et al., 2011), suggesting that IL‐6 and other myokines could have a direct role in reducing cancer progression by modulating key signaling pathways involved in cancer cell proliferation. For instance, murine studies show that aerobic exercise regulates the Protein Kinase B/mammalian target of rapamycin (AKT/mTOR) signaling pathway (Thompson et al., 2009) which is a key cell growth regulator and is hyperactivated in many cancer types (Porta et al., 2014). While the exact mechanisms underlying the exercise‐dependent deactivation of AKT/mTOR signaling have not been fully elucidated, IL‐6 is a potential candidate because of its effects on AMP‐activated protein kinase (AMPK) signaling. IL‐6 release from skeletal muscle during exercise is known to activate AMPK (MacDonald et al., 2003), which can inhibit mTOR and its downstream effectors such as ribosomal protein S6 kinase beta‐1 (S6K1) through a Tuberous Sclerosis Complex 2 (TSC2)‐dependent or ‐independent mechanism (Bolster et al., 2002; Kawaguchi et al., 2015). Kurgan et al. (2017) showed that incubating A549 nonsmall cell lung cancer cells with exercise‐conditioned serum reduced cell growth and decreased the phosphorylation levels of AKT, mTOR, and S6K1. However, exercise‐conditioned serum did not influence the growth of MRC5 normal lung fibroblasts (Kurgan et al., 2017), suggesting that the effect of exercise serum may be specific to malignant cells that harbor aberrant activation of signaling pathways. Interestingly, incubation with serum collected 24‐hr after exercise cessation also reduced A459 cell growth and inhibited the activation of these signaling molecules (Kurgan et al., 2017). It is well recognized that most serum markers elevated during acute exercise, including IL‐6 and other myokines, return to basal levels within 24 hr of exercise cessation (Cerqueira et al., 2019). Thus, it is unclear whether IL‐6 directly contributed to the prolonged inhibition of the AKT/mTOR signaling pathway in this study, or whether downstream priming effects were involved.

Dethlefsen, Hansen, et al. (2017) also showed that serum obtained after combined resistance and aerobic interval exercise activated the Hippo signaling pathway, which involves a kinase cascade from the tumor suppressors mammalian Mst1 and Mst2 to the oncoproteins Yes‐associated protein (YAP) and transcriptional coactivator with PDZ‐binding motif (TAZ) (Pan, 2010). Stimulating MCF‐7 cells with postexercise serum induced the phosphorylation and subsequent cytosolic sequestration of YAP, which was associated with a reduced expression of genes downstream of YAP (e.g., ANKRD1) (Dethlefsen, Hansen, et al., 2017). Direct stimulation of breast cancer cells with epinephrine/norepinephrine mimicked this effect and combining participant serum with a β‐adrenergic blocker (propranolol) decreased the phosphorylation of YAP, suggesting adrenergic signaling was responsible for the exercise‐induced suppression of cancer cell growth (Dethlefsen, Hansen, et al., 2017). However, the same study showed no significant effect of exercise‐conditioned serum on total or phosphorylated levels of YAP in MDA‐MB‐231 cells (Dethlefsen, Hansen, et al., 2017). More recently, Baldelli et al. (2020) also showed no significant effect of serum collected immediately postexercise on YAP phosphorylation in MDA‐MB‐231 or LNCaP cells. Taken together, it is uncertain from the current evidence whether the ability of exercise to modulate downstream effectors of the Hippo signaling pathway contributes to the antimitotic effect of exercise.

There were concerns regarding the risk of bias within individual studies. Common issues included unclear fidelity to the exercise protocol, a lack of assessor blinding, and inadequate control of potentially confounding variables such as diet and physical activity prior to the experimental session. Inadequate control of diet may affect the serological response to exercise. For example, depletion of intramuscular glycogen stimulates enhanced release of muscle‐derived IL‐6 (Fischer, 2006) and therefore consuming a high carbohydrate meal prior to exercise may attenuate IL‐6 release compared with a lower carbohydrate meal. Dietary bioactive compounds and antioxidant capacity may also influence serum‐stimulated cancer cell growth (Fleshner et al., 1999; Tymchuk et al., 2001). Furthermore, despite that our eligibility criteria only required studies to have obtained a blood sample before and immediately after an acute exercise bout, no included study attempted to control for measurement error and/or normal biological variability in serum markers by employing a randomized, controlled, crossover study design. Indeed, plasma cytokine concentrations (IL‐6 and IL‐10) have been shown to increase from pre to postexercise, but this increase was not significantly different from a nonexercise control (Windsor et al., 2018), highlighting the importance of including a control condition. As well as bias within individual studies, Egger's test of the intercept and visual inspection of the funnel plot were suggestive of small‐study effects, which may indicate publication and/or reporting bias, among other issues (Page et al., 2020). Thus, these potential biases should be considered when interpreting the findings of this meta‐analysis, and further trials using more rigorous experimental designs are warranted.

There was evidence of considerable heterogeneity between studies. The inclusion of study characteristics as covariates in the meta‐analytic model, including participant age and disease status, exercise type, cancer type, cell line TP53 status, and serum incubation time, had a negligible influence on the magnitude of heterogeneity or the pooled treatment effect. However, it is unlikely that the number of trials included in the meta‐regressions was sufficient to provide adequate statistical power to detect moderating effects, and the results should be treated with caution. Because differences in subgroups observed within studies are more reliable than analyses of subsets of studies (Deeks et al., 2019), further research should directly evaluate whether the antimitotic effect of exercise is modified by these factors. For instance, given the important role of intensity on the serological response to exercise (Fischer, 2006; Zouhal et al., 2008) a direct comparison of the effects of work‐matched moderate‐ versus high‐intensity aerobic exercise on serum‐stimulated cancer cell growth is warranted. Hwang et al. (2020) did show a statistical reduction in LNCaP cell growth in older but not younger adults following stimulation with postexercise compared with preexercise serum, suggesting a modifying effect of age. However, the magnitude of effects was not directly compared between the two subgroups, which is required for valid inference (Deeks et al., 2019; Gelman & Stern, 2006).

A limitation of all studies included in this review is the use of two‐dimension (2D) cell culture models, which do not fully mimic the in vivo tumor morphology or microenvironment. Another drawback to cells growing in a 2D monolayer is the unlimited access to essential compounds in the culture medium such as oxygen, nutrients, and serum (Kapałczyńska et al., 2018). This is in contrast to cancer cells in vivo, which have variable access to oxygen and nutrients due to the architecture of the tumor mass (Kapałczyńska et al., 2018). Three‐dimensional (3D) cell cultures better recapitulate in vivo tumor physiology and the physical characterizes of a solid tumor mass (Duval et al., 2017). Two studies included in this review used 3D cell culture techniques to show that exercise‐conditioned serum reduces the clonogenic potential of breast (Baldelli et al., 2020; De Santi et al., 2019) and prostate (Baldelli et al., 2020) cancer cells. Future research should consider using 3D cell culture models, such as patient‐derived tumor spheroids, to better reflect in vivo tumor conditions and provide stronger evidence on the effect of acute exercise on cancer cell growth. Future studies should also consider controlling for hydration status because hypohydration may increase serum protein concentration, which could impact serum‐stimulated cell growth.

It is worth noting that we limited our review to studies that collected serum within 5 min of acute exercise cessation. This was to standardized the timepoint at which sera was sampled because this can have a substantial effect on the serological response (Brooks et al., 1990), and to ensure we did not include studies that characterized serological changes in response to a chronic exercise intervention (as opposed to acute exercise). Nevertheless, some studies have shown that serum collected up to 24 hr postacute exercise inhibits cancer cell growth (Baldelli et al., 2020; Kurgan et al., 2017; Rundqvist et al., 2013), suggesting that the serological response to acute exercise may have growth inhibitory effects for several hours after exercise cessation.

In conclusion, we showed that exercise‐conditioned serum significantly reduces in vitro cancer cell growth. This finding provides preliminary evidence that acute exercise‐induced modulations in serum markers may, at least in part, explain the link between regular physical activity and reduced cancer risk. Many questions still remain regarding the underlying mechanistic pathways and potential effect modifiers such as exercise intensity and cancer cell phenotype. To strengthen this evidence‐base, future studies should seek to reduce the risk of bias by using more rigorous experimental designs and consider using 3D cell culture models to better replicate in vivo tumor conditions.

## CONFLICT OF INTEREST

The authors have no conflicts of interest to declare.

## AUTHOR CONTRIBUTIONS

All authors were responsible for the conceptualization and formulation of research objectives. STO and JMS were responsible for developing the systematic review protocol. STO preregistered the protocol, performed the systematic searches, removed duplicates, and screened the titles and abstracts. STO and ARJ assessed full‐texts for eligibility, and all authors confirmed the eligibility of each included study. STO and ARJ extracted data from eligible studies, and all authors were involved in the risk of bias assessment. STO was responsible for data curation, statistical analyses, data presentation, and writing the original draft. All authors were involved in revising the manuscript and approved its submission.

## ETHICAL STATEMENT

Ethics approval was not required to undertake this systematic review.

## Data Availability

The search results, dataset, statistical code, and detailed risk of bias assessment from this review are available in the Open Science Framework repository: https://osf.io/e5vj8/.
